# Editorial: Recent advances in molecular properties of DNA-protein interactions, chromatin and their biological roles

**DOI:** 10.3389/fmolb.2023.1171714

**Published:** 2023-03-07

**Authors:** Xiangyan Shi, Lars Nordenskiöld, Olga S. Sokolova, Alexey K. Shaytan

**Affiliations:** ^1^ Department of Biology, Shenzhen MSU-BIT University, Shenzhen, Guangdong, China; ^2^ School of Biological Sciences, Nanyang Technological University, Singapore, Singapore; ^3^ Department of Biology, Lomonosov Moscow State University, Moscow, Russia

**Keywords:** chromatin, structure, dynamics, DNA-protein interactions, cryo-EM, NMR, dead-Cas-systems

DNA-protein interactions form the basis of genome functioning. Billions of years of evolution have tailored specific patterns of protein-DNA interactions and their conformational and dynamical properties to allow for complex regulation of gene networks and their responses to internal and external stimuli. Deciphering these complex regulatory mechanisms is a major challenge for the biological sciences in the twenty-first century. This Research Topic presents papers that shed light on various aspects of chromatin biology.

Understanding the genome functioning in eukaryotic organisms depends on our knowledge of chromatin structure and dynamics. Chromatin modulation factors regulate the biological activities of DNA through introducing changes to the molecular properties of the DNA-protein complex. Remarkable progress has been recently achieved in delineating the molecular properties in chromatin regulation with the technical advances in cryo-EM (Wilson and Costa, 2017; Takizawa and Kurumizaka, 2022), NMR (Kitevski-LeBlanc et al., 2018; Shi et al., 2018; Xiang et al., 2018; Morrison et al., 2021), MD (Armeev et al., 2021; Farr et al., 2021; Micheletti et al., 2021) and other high spatial and temporal resolution methods. Solid-state NMR (SSNMR) has been emerging as a primary technique to determine the structure, internal dynamics, phase separation mechanisms and nucleosome-protein interactions in chromatin regulation (Shi et al., 2018; Xiang et al., 2018; Sanulli et al., 2019; Shi et al., 2020; Ackermann and Debelouchina, 2021), particularly, it enables to track the dynamics and conformation at the atomic resolution for both flexible and rigid regions of nucleosomes. Conventional ^13^C-detected SSNMR and, recently, ^1^H-detected ultra-fast MAS SSNMR have been implemented to study nucleosomes and revealed unique functional relevant conformation and dynamics of histones in chromatin. Smrt et al. performed a ^1^H-detected SSNMR study on 16-mer nucleosome arrays with different linker DNA lengths, and compared the conformations of H3 in the nucleosome arrays. The high quality of the multidimensional ^1^H-detected dipolar-based SSNMR spectra allows the nearly complete backbone resonance assignments for H3 globular domain. The comparison of H3 chemical shift differences among the nucleosome arrays with different linker DNA lengths explored no major structural changes and only local conformational differences between them in a number of domains, of which many residues are involved in key histone-DNA interactions and impacting nucleosome stability. This study provides another example of the power of SSNMR to determine the unique molecular properties of compacted nucleosomes and nucleosome arrays modeling chromatin at close to physiological conditions.

Cryo-EM has been a major driver in deciphering the structure of chromatin complexes recently. A comprehensive analysis of all the structures of nucleosomes and their complexes deposited in PDB is presented by Armeev et al. More than 60 new structures have been deposited in 2022. However, approaches that integrate different experimental methods and modeling can give even more insights. For example, as part of this Research Topic, a study presents 3D structural study of an unwrapping the nucleosome by human FACT (FAcilitates Chromatin Transcription) (Volokh et al.), a multifunctional histone chaperone involved in transcription, replication and DNA repair (Valieva et al., 2016; Sivkina et al., 2022). Recently, a class of anti-cancer agents (curaxins) that disturbs DNA/histone interactions within nucleosomes has been characterized (Kantidze et al., 2019). Volokh et al. analyzed the effects of curaxin CBL0137 on nucleosome unfolding by FACT using a combination of spFRET, molecular modeling and electron microscopy. This approach allowed us to build the model of FACT-dependent nucleosome unfolding taking into account the importance of forming the “decision point” structural intermediates.

Onufriev proposes an interesting approach to quantify the experimentally known destabilization effect on protein or protein/DNA complexes due to increase in intracellular pH. The study suggests quantifying the effect by considering ∆∆G0.3; meaning the change in the binding free energy due to a pH increase of 0.3 units, corresponding to a doubling of the H^+^ activity, since such magnitude of changes are typical during various cellular processes. The calculations use publicly available software to compute protein aa pK values in combination with an in-house script for nucleic acid-protein complexes. In particular the author considers the consequences of such pH changes for the stability and unwrapping of DNA from the histone octamer in the nucleosome and its relation to processes that require DNA unwrapping from the histone octamer in the nucleus. Onufriev suggests a threshold of ½(k_B_T) for changes in the stability of nucleosomal protein-DNA complexes to be biologically significant. The author surmises that such a change in the binding affinity above the threshold could significantly destabilize the nucleosomal DNA-histone octamer complex. The computations are based on continuum electrostatics Poisson-Boltzmann approach and subsequent calculations of free energy differences.

Despite the progress in understanding the properties and dynamics of chromatin elements *in vitro*, the ultimate goal is to understand how chromatin functions *in vivo*, and moreover, how gene regulatory networks operate at the functional level. The complexity of these networks does not suggest that we will be able to understand their overall functioning simply by studying the individual protein-protein and protein-DNA interactions. An alternative approach would be to perturb the functioning of the natural gene regulatory networks and analyze their response, or ultimately to try to create artificial regulatory networks and learn from this process. An enormous leap forward in our ability to targetedly influence the functioning of gene regulatory networks has been established through the use of catalytically inactive CRISPR-Cas systems, the so-call, dead-Cas-systems, or dCas (Xu and Qi, 2019). The complex of dCas protein with its sgRNA can be used to influence transcription at specific genomic loci and used to deliver different linked chromatin and epigenetic effectors to target sites. A mini-review highlighting recent advances in such technologies, particularly in creation of artificial gene circuits and challenges ahead is also present in this Research Topic (Shaytan et al., 2022).

In conclusion, chromatin is a dynamic structure consisting of DNA and proteins, and its dynamic nature makes its structural studies very complex. The need to continue developing new comprehensive approaches for the study of chromatin is undeniable.
